# Bilateral Decompressive Craniectomy for Subdural Empyema: A Case Report

**DOI:** 10.7759/cureus.105926

**Published:** 2026-03-26

**Authors:** Georgi Krasimirov Georgiev, Nurfet Alioski, Tihomir Eftimov, Todor Shamov

**Affiliations:** 1 Neurosurgery, Saint Anna University Hospital, Sofia, BGR

**Keywords:** bilateral craniectomy, cranial abscess, cranial subdural empyema, decompressive craniectomy, subdural

## Abstract

Intracranial subdural empyema (SDE) is a rare but life-threatening condition characterized by the rapid spread of purulent infection within the subdural space and a high risk of neurological deterioration. Despite significant improvements in neuroimaging, antimicrobial therapy, and surgical techniques, severe cases may progress to diffuse cerebral edema and refractory intracranial hypertension, challenging conventional management strategies. Surgical evacuation remains the cornerstone of treatment; however, the role of decompressive craniectomy in infection-related intracranial hypertension is not well established and is supported primarily by isolated reports. We present a case of fulminant SDE requiring bilateral decompressive craniectomy as a life-saving measure due to uncontrollable intracranial pressure. This case underscores the potential role of aggressive decompressive surgery in selected patients with severe inflammatory brain swelling secondary to intracranial infection and highlights the need for heightened clinical awareness and individualized surgical decision-making. While bilateral decompressive craniectomy remains exceedingly rare in the context of SDE, this report contributes to the limited body of literature and suggests that such an approach may be considered in extreme cases when standard surgical interventions fail to achieve adequate intracranial pressure control.

## Introduction

Intracranial subdural empyema (SDE) is defined as a purulent collection located between the dura mater and the arachnoid mater and represents one of the most aggressive forms of intracranial infection due to its capacity for rapid spread along the subdural space in the absence of anatomical barriers [1]. This unrestricted dissemination over the cerebral convexities and along the falx cerebri predisposes patients to early mass effect, venous thrombosis, cortical irritation, and abrupt neurological deterioration, thereby establishing SDE as a neurosurgical emergency requiring prompt diagnosis and intervention [1,2].

Historically, SDE was associated with extremely high mortality. In the pre-antibiotic and early antibiotic eras, mortality rates reached approximately 42% in the 1950s, reflecting delayed diagnosis and limited surgical and antimicrobial options [2,3]. With the introduction of modern neuroimaging, broad-spectrum antibiotics, and improved neurosurgical techniques, mortality progressively declined to around 10% by the 1990s, and further reductions have been reported in contemporary series [2,4]. Despite these improvements, SDE continues to carry a substantial risk of long-term neurological morbidity [2,5].

Surgical treatment remains a cornerstone of SDE management. Standard approaches include burr-hole drainage or craniotomy for evacuation of purulent collections, combined with prolonged intravenous antibiotic therapy [2,4]. In cases complicated by severe cerebral edema and refractory intracranial hypertension, decompressive craniectomy may be required as a life-saving measure, particularly in pediatric and young adult populations [5,6,7]. 

Precise data regarding the frequency of decompressive craniectomy in SDE are limited, as most evidence derives from case reports and small series rather than large cohort studies [8,9]. Nevertheless, available reports suggest that decompressive craniectomy may play a critical adjunctive role when conventional empyema evacuation fails to control intracranial pressure [5-10]. Bilateral decompressive craniectomy for cranial infection diseases is exceedingly rare and is generally reserved for fulminant cases with diffuse cerebral swelling, such as meningitis and encephalitis. However, based on our research, this is the first published case of bilateral decompressive craniectomy for SDE [9,10].

## Case presentation

A 48-year-old female patient was admitted to our hospital in the Infectious Disease Department late at night with acute neurological deterioration manifested by progressive confusion, somnolence, and marked psychomotor slowing. The Glasgow Coma Scale (GCS) score was 12 with positive Brudzinski and Kernig signs. According to the information provided by her relatives, effective communication with the patient had become increasingly difficult over the preceding 48 hours. During this period, she had complained of diffuse headache accompanied by febrile episodes, with body temperature reaching approximately 38°C. The patient had been previously treated for sinusitis; however, reliable information regarding the etiological agent, duration, and adequacy of antimicrobial therapy could not be obtained.

**Figure 1:**
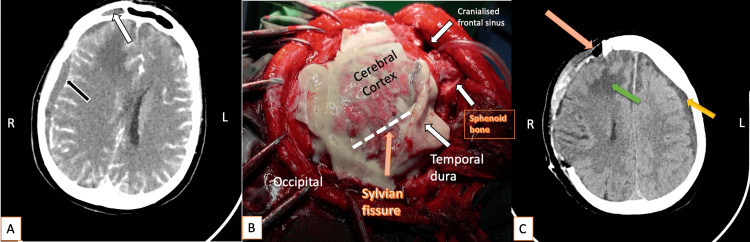
(A) Preoperative contrast-enhanced CT demonstrates a right-sided subdural collection (black arrow) and a collection within the right frontal sinus (white arrow). (B) Intraoperative findings reveal purulent material following cruciate dural opening, with hyperemic cortical surface and encephalitic changes. The Sylvian fissure is indicated by an orange arrow, and the inferiorly displaced temporal dura by a white arrow. (C) Early postoperative CT (third postoperative day) shows frontal sinus cranialization (beige arrow), CT signs of early frontal cerebritis (green arrow), marked cerebral edema, and a contralateral subdural collection measuring approximately 8 mm (yellow arrow), with restored midline alignment. CT = computed tomography; L = left; R = right

Due to progressive worsening of the patient’s condition with a GCS score of 10 and left central hemiparesis, a neurosurgical consultation was requested. Contrast-enhanced computed tomography (CT) revealed the presence of a subdural fluid collection with radiological features suggestive of an infectious process. Laboratory evaluation revealed a marked inflammatory response. On admission, the patient demonstrated severe leukocytosis with granulocytosis, with a total white blood cell count of 36 × 10⁹/L (reference range: 3.5-10.5 × 10⁹/L) and an elevated granulocyte count of 32.3 × 10⁹/L (reference range: 3.5-5.5 × 10⁹/L). C-reactive protein levels exceeded 200 mg/L (reference: <5 mg/L), and fibrinogen was markedly elevated at 7.73 g/L (reference range: 2.0-4.5 g/L) (Table 1). Based on the imaging findings (Figure 1, Panel A) and clinical presentation, the most likely diagnosis was SDE.

**Table 1:** Laboratory findings indicate a pronounced systemic inflammatory response at presentation, characterized by marked leukocytosis with granulocytosis and substantially elevated acute-phase reactants, including C-reactive protein and fibrinogen, consistent with severe intracranial infection.

Parameter	Patient value	Reference range (units)
White blood cell count	36 × 10⁹/L	3.5–10.5 × 10⁹/L
Granulocyte count	32.3 × 10⁹/L	3.5–5.5 × 10⁹/L
C-reactive protein	>200 mg/L	<5 mg/L
Fibrinogen	7.73 g/L	2.0–4.5 g/L

Given the acute neurological deterioration and radiological evidence of intracranial infection, the patient underwent emergency surgical intervention. A decompressive craniectomy was performed, primarily indicated by the presence of pronounced cerebral edema observed intraoperatively, which required adequate decompression to prevent further intracranial hypertension and secondary brain injury. Evacuation of the subdural collection was performed, and purulent material was identified (Figure 1, Panel B). Intraoperative samples were obtained for microbiological analysis. Before surgery, additional microbiological specimens had been collected, including a throat swab, a urine sample, and blood cultures.

During the same surgical procedure, exploration of the frontal sinus revealed a defect of the frontal sinus wall with direct communication between the sinus cavity and the epidural-subdural space. This finding was considered the most probable source of the intracranial infection. Consequently, cranialization of the frontal sinus was performed, including complete evacuation of purulent contents from the sinus cavity (Figure 1, Panel C). Reconstruction was achieved using standard surgical techniques, involving obliteration of the sinus with autologous muscle tissue and sealing with fibrin glue to ensure secure separation between the sinus and intracranial compartments. Calvaria from decompressive craniectomy was placed in the abdominal cavity in the right hypogastric area.

**Figure 2:**
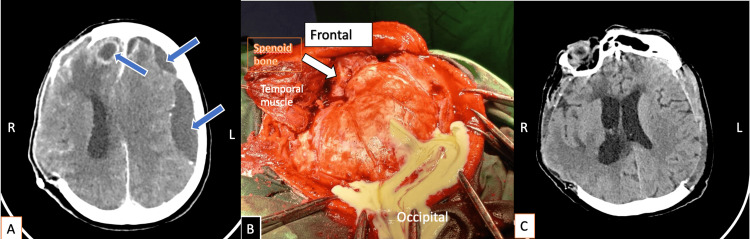
(A) Contrast-enhanced postoperative CT performed two to three days after the first surgery demonstrates enlarging subdural collections over the left hemisphere, predominantly along the convexity, with the largest collection in the fronto-temporo-parietal region and several smaller frontal collections. An associated left frontal intracerebral abscess with features of evolving cerebritis is indicated (blue arrow). (B) Intraoperative findings show purulent discharge following minimal dural incision during wide decompressive craniectomy. (C) Postoperative imaging demonstrates the result of bilateral wide decompressive craniectomy, with persistent cerebral edema but near-complete resolution of the mass effect and midline shift. CT = computed tomography; L = left; R = right

Postoperatively, the patient initially demonstrated clinical improvement. She was successfully extubated and showed neurological recovery, reaching a GCS score of approximately 14 points. However, two days after the initial intervention, the patient experienced renewed neurological deterioration, with progressive decline in consciousness and a reduction of the GCS score to 8 points and right hemiparesis. A control CT scan revealed newly developed epidural and subdural collections located contralaterally to the initial surgical site (Figure 2, Panel A). In light of the clinical and radiological findings, an urgent contralateral decompressive craniectomy was performed, aimed at evacuation of the pathological collections and control of intracranial pressure (Figure 2, Panel B).

Following the second surgical intervention, the patient was managed in the intensive care unit for three days, during which gradual neurological stabilization was achieved, accompanied by resolution of the inflammatory process. Subsequently, she was transferred to the neurosurgical ward in stable general condition with a good postoperative CT result (Figure 2, Panel C).

**Figure 3:**
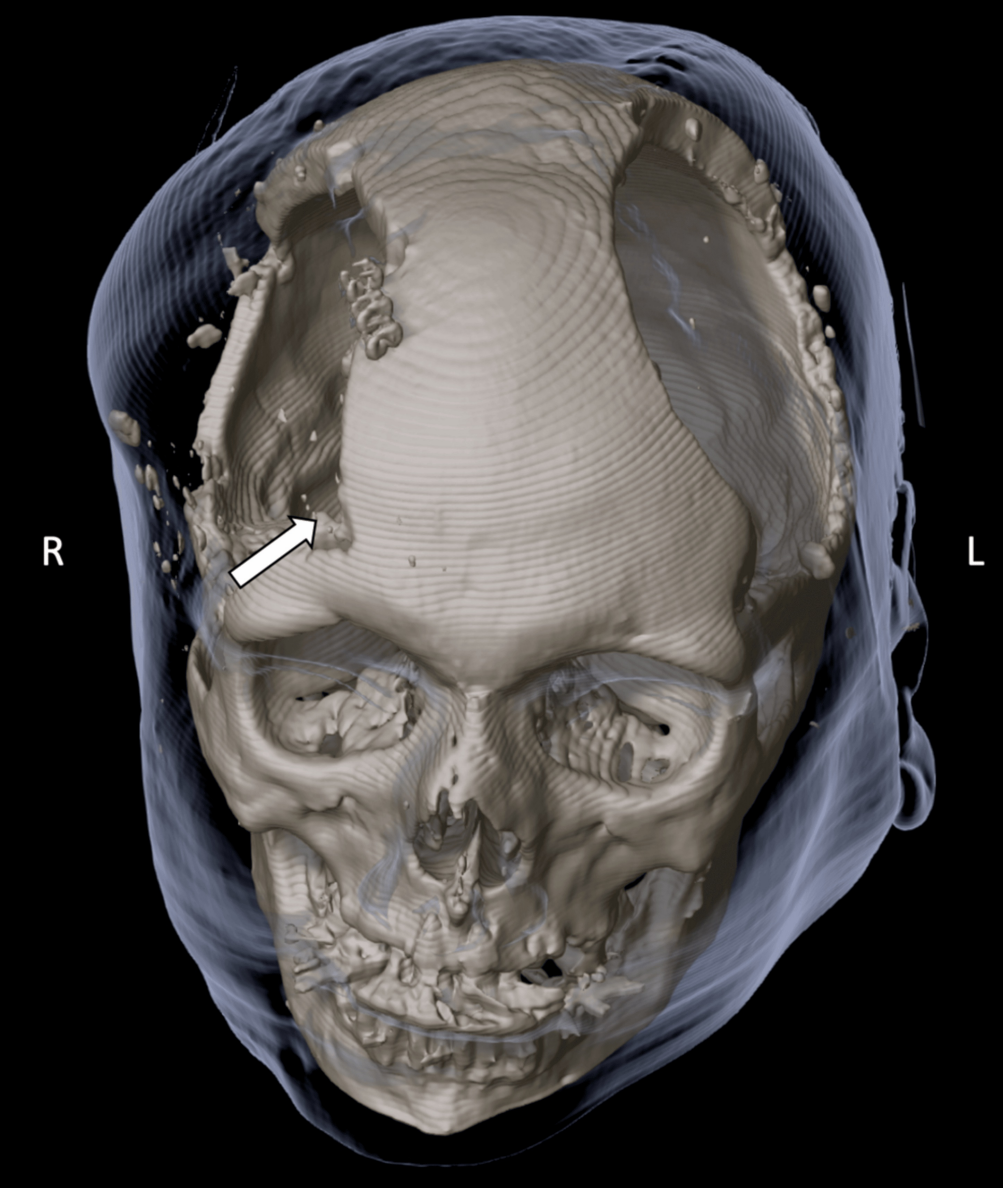
Three-dimensional CT reconstruction illustrating the extent of the bilateral decompressive craniectomies one day before hospital discharge. Soft tissues are visualized using a gray rendering. The cranialization of the frontal sinus is indicated by a white arrow. CT = computed tomography; L = left; R = right

A broad-spectrum antimicrobial regimen was initiated due to suspected intracranial infection. During the acute postoperative phase, the patient received empirical therapy of ceftriaxone, vancomycin, and metronidazole. Based on microbiological findings, *Klebsiella pneumoniae* and *Klebsiella oxytoca* from a throat swab, *Staphylococcus epidermidis* from blood cultures, and *Proteus mirabilis* from urine, the regimen was revised to linezolid and imipenem. *Klebsiella pneumoniae* and *Proteus mirabilis* were detected on the 20th and 25th day of the hospital stay, with a likely nosocomial etiology. Despite these findings, cultures from intraoperatively obtained purulent material remained negative. Residual soft-tissue edema was observed on CT the day before discharge (Figure 3). After discharge, the antimicrobial therapy was continued with linezolid for four weeks and rifampicin for up to three months.

**Figure 4:**
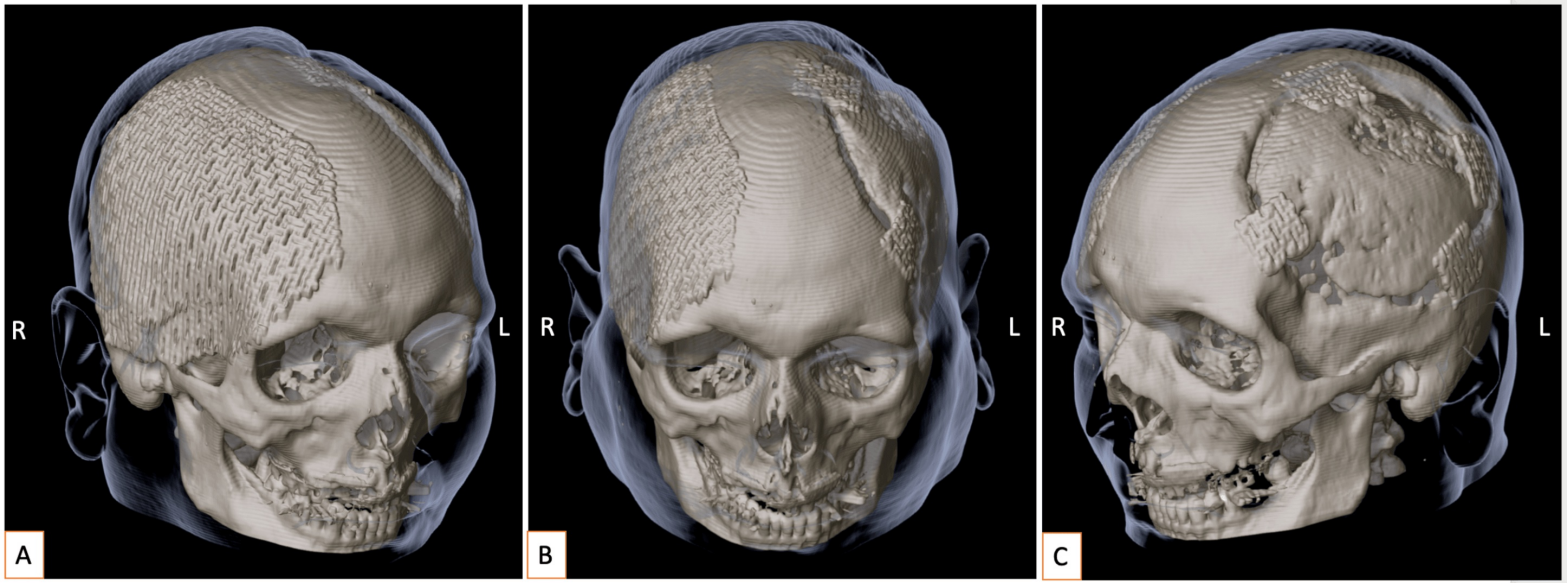
Postoperative outcome one month after the final surgical intervention. (A) Right-sided view with the head rotated to the left, demonstrating reconstruction of the decompressive craniectomy defect using a titanium plate. (B) Frontal view illustrating the overall result of both surgical procedures. (C) Left-sided view with the head rotated to the right, showing reconstruction of the cranial defect using the patient’s own bone flap. Early signs of bone flap resorption are evident, necessitating fixation with small titanium plates. Soft tissue contours are visualized in grey contour, demonstrating a satisfactory cosmetic outcome. L = left; R = right

After complete resolution of the acute infectious process, the patient was readmitted three months later for staged reconstructive surgery. Cranioplasty was initially performed to restore the cranial defect on the right side. One month later, sinking bone flap syndrome was noted. Reconstruction of the left-sided cranial defect was performed using calvaria placed in the subcutaneous soft tissue of the abdominal cavity. Due to initial bone resorption processes, additional titanium plaque was used for fixation. The final postoperative outcome of the cranial reconstruction is demonstrated in the three-dimensional CT reconstruction (Figure 4).

Long-term follow-up over a period of two years demonstrated a favorable clinical outcome. The patient remained in good general condition, without evidence of recurrent infection, febrile episodes, or chronic inflammatory changes involving the frontal sinus or otorhinolaryngological structures. No significant neurological deficits were observed, with the exception of intermittent headaches. The patient also reported mild residual cognitive disturbances, predominantly affecting attention and short-term memory. These symptoms gradually improved under supportive neurostimulators and gastroprotective and pharmacological therapy, and had almost completely resolved at the 2.5-year follow-up. At the most recent evaluation, the patient was fully conscious, neurologically stable, afebrile, and without clinical or radiological signs of persistent or recurrent sinus-related or intracranial inflammatory pathology.

## Discussion

The most common source of infection for SDE is paranasal sinusitis, particularly of the frontal sinus, followed by otogenic infections, meningitis, cranial trauma, dental infections, and neurosurgical procedures [7]. Sinogenic SDE typically arises from frontal sinusitis with subsequent intracranial spread, leading to meningitis, SDE, intracerebral abscess, osteomyelitis, or their combination [7,8]. Approximately 0.1% of complicated sinusitis cases develop intracranial extension, presenting as SDE, intracerebral collections, cranial osteomyelitis, or mixed infections [7]. Rapid dissemination of purulent material along the cerebral convexities and interhemispheric space results in mass effect, venous thrombosis, and neurological deterioration, making SDE a neurosurgical emergency requiring urgent intervention [2,7].

Epidemiologically, SDE is rare, and precise incidence estimates are limited by the predominance of retrospective series and the frequent conflation of empyema with brain abscesses in national datasets [2,9,11]. One of the most robust population-based estimates derives from a 10-year multicenter study in Queensland, Australia, reporting an incidence of approximately 0.1 per 100,000 persons for intracranial SDE [11]. Other reviews cite incidence ranges of 0.2-1.9 per 100,000, though these figures stem from heterogeneous cohorts with variable definitions and should be interpreted cautiously [3,12]. In pediatric populations, combined intracranial empyema (epidural and subdural) rates of 2.8-5.7 per million children per year have been reported, highlighting the rarity of the condition among children and adolescents [13].

Age distribution for SDE often shows peaks in children/adolescents and young adults, typically associated with contiguous sinonasal or otogenic infection, whereas older adults are more frequently represented in postoperative or trauma-related cases [2,4,5,13]. Male predominance has been described across many series, though the magnitude varies with demographic and etiologic factors [2,13].

Pathological mechanism of development

From a microbiological standpoint, approximately 20% of SDE cases are culture-negative, most often due to prior antibiotic therapy or difficulties in isolating fastidious organisms [2,7]. Polymicrobial infections occur in about 14% of cases, particularly in sinogenic and odontogenic origins [7]. Streptococci, especially the *Streptococcus anginosus* (*milleri*) group, and anaerobes predominate in sinus-related SDE, whereas *Staphylococcus aureus* is more common in postoperative and trauma-associated cases [2,4,5].

Up to 20% of intracranial infections are estimated to occur in immunocompromised or immunologically altered patients, which is associated with atypical presentations and more rapid progression, particularly in the context of chronic disease or recent surgery [12,13].

Notably, frontal SDE does not always correlate with overt purulence in the frontal sinus. The most likely mechanism is retrograde thrombophlebitis of valveless diploic veins, enabling hematogenous spread to the subdural space [2]. Consequently, intracranial empyema may develop despite minimal or absent radiological sinus findings, necessitating a high index of clinical suspicion [2].

Clinical presentation of subdural empyema

Clinically, SDE presents with nonspecific features such as headache, fever, altered mental status, focal deficits, and seizures [2,5]. Early symptoms may mimic sinusitis or mild infection, often delaying diagnosis and worsening outcomes [4,14]. Surgical management is essential and includes empyema evacuation, debridement, and cranial opening procedures, selected based on lesion size, location, and cerebral edema [2,13]. In a cohort of 90 cases, 44.4% underwent craniotomy rather than burr-hole drainage, showing improved radiological clearance and clinical response, although overall outcomes were comparable [4].

In cases complicated by refractory intracranial hypertension, decompressive craniectomy may be indicated [2,14]. This procedure allows brain expansion through skull removal, reducing intracranial pressure and secondary injury [14]. While primarily established in traumatic brain injury and malignant stroke, decompressive craniectomy has been reported in SDE when standard evacuation fails to control intracranial pressure. Available evidence highlights its role as a salvage option to prevent cerebral hypoperfusion and herniation, although data in infectious contexts remain limited [5,9,10].

Subdural empyema and decompressive craniectomy

A notable case report described the successful bifrontal decompressive craniectomy for acute SDE in a 13-year-old with sinusitis-associated empyema presenting with catastrophic intracranial hypertension, highlighting the role of wide decompression in selected severe cases [9]. However, such reports remain limited to individual case experiences rather than cohort studies, and comprehensive incidence data specific to the use of decompressive craniectomy for SDE are not available in large series. The absence of larger systematic data means that precise frequency estimates of decompressive craniectomy in the setting of SDE or bilateral decompressive craniectomy for SDE cannot be reliably derived from existing literature; rather, the literature consists primarily of case-based evidence that illustrates potential application in severe or refractory presentations [3,5,9,10,13].

Outcomes after SDE depend on multiple factors, including timely diagnosis, efficacy of surgical drainage, control of intracranial pressure, and appropriate antimicrobial therapy [4,5]. Mortality in modern series is markedly lower than historical reports, often in single digits, though neurological sequelae, including persistent focal deficits and seizures, are common among survivors [2,5,13]. The integration of decompressive craniectomy into the surgical strategy for SDE with severe intracranial hypertension is informed by case reports demonstrating feasibility and potential benefit, but large-scale data are lacking, and such interventions should be considered on a case-by-case basis within multidisciplinary care frameworks.

In our opinion, decompressive craniectomy should be considered a crucial therapeutic option in carefully selected cases of SDE, as it may be life-saving. The rationale for this approach is well illustrated by our case, in which marked cerebral edema and subdural collection were present with clinical correspondence of hemiparesis and low GCS. By this stage, we managed SDE based on subacute or acute subdural hematoma collection management requirements. In our experience, the possibility of nasal spreading infection needs a craniotomy or even a decompressive craniectomy due to remaining possible pathological fistulae. Under such circumstances, management with burr-hole drainage alone may precipitate a sudden increase in intracranial pressure, thereby significantly increasing the risk of secondary brain injury and transtentorial herniation. The second decompressive craniectomy was performed using the same principles based on mass-effect collection, pseudoherniation, and rapid neurological deterioration.

In addition, the coexistence of intraparenchymal infectious foci, such as brain abscesses or areas of early or late cerebritis, as demonstrated on CT, further compromises cerebral compliance and limits the brain’s capacity to tolerate even minor fluctuations in intracranial volume. Consequently, simple evacuation of the subdural collection may be insufficient and potentially hazardous.

For these reasons, we believe that decompressive craniectomy, particularly in patients with severe cerebral edema and associated parenchymal involvement, should be taken into consideration as part of the surgical strategy. In selected cases, this approach may represent the critical factor determining survival versus fatal neurological deterioration.

The present case highlights the feasibility of decompressive craniectomy as a life-saving intervention in fulminant SDE when conventional surgical evacuation alone is inadequate. The pathophysiological rationale for such an approach lies in the diffuse inflammatory cerebral swelling and venous outflow compromise that can accompany SDE, leading to global intracranial pressure elevation rather than focal mass effect. Although bilateral decompression is rarely reported in infectious neurosurgical pathology, this case suggests that, in selected patients, aggressive surgical decompression may be justified to prevent secondary brain injury. Given the paucity of data and the absence of standardized guidelines, further accumulation of similar cases is necessary to better define indications, timing, and outcomes of decompressive craniectomy, particularly bilateral procedures, in the management of severe SDE.

## Conclusions

Intracranial SDE continues to represent a neurosurgical emergency associated with significant morbidity despite modern diagnostic and therapeutic advances. This case illustrates that bilateral decompressive craniectomy may be a viable salvage option in exceptionally severe presentations characterized by diffuse cerebral edema and uncontrollable intracranial hypertension. Although such an approach should remain highly selective, it expands the surgical armamentarium for managing fulminant SDE and underscores the need for individualized, pathophysiology-driven decision-making in complex infectious neurosurgical cases.
